# Electrophysiological Mechanisms of Gastrointestinal Arrhythmogenesis: Lessons from the Heart

**DOI:** 10.3389/fphys.2016.00230

**Published:** 2016-06-14

**Authors:** Gary Tse, Eric T. H. Lai, Alex P. W. Lee, Bryan P. Yan, Sunny H. Wong

**Affiliations:** ^1^Li Ka Shing Faculty of Medicine, School of Biomedical Sciences, The University of Hong KongHong Kong, China; ^2^Department of Medicine and Therapeutics, The Chinese University of Hong KongHong Kong, China; ^3^Department of Medicine and Therapeutics, Institute of Digestive Disease, Li Ka Shing Institute of Health Sciences, The Chinese University of Hong KongHong Kong, China

**Keywords:** gastrointestinal electrophysiology, cardiac electrophysiology, electrical excitation, arrhythmia, focal activity, reentry

## Abstract

Disruptions in the orderly activation and recovery of electrical excitation traveling through the heart and the gastrointestinal (GI) tract can lead to arrhythmogenesis. For example, cardiac arrhythmias predispose to thromboembolic events resulting in cerebrovascular accidents and myocardial infarction, and to sudden cardiac death. By contrast, arrhythmias in the GI tract are usually not life-threatening and much less well characterized. However, they have been implicated in the pathogenesis of a number of GI motility disorders, including gastroparesis, dyspepsia, irritable bowel syndrome, mesenteric ischaemia, Hirschsprung disease, slow transit constipation, all of which are associated with significant morbidity. Both cardiac and gastrointestinal arrhythmias can broadly be divided into non-reentrant and reentrant activity. The aim of this paper is to compare and contrast the mechanisms underlying arrhythmogenesis in both systems to provide insight into the pathogenesis of GI motility disorders and potential molecular targets for future therapy.

## Introduction

Abnormalities in the orderly activation and recovery of impulses traveling through the heart and the gastrointestinal (GI) tract can lead to arrhythmogenesis (Tse, 2015; Tse and Yeo, 2015; Tse et al., 2016k). Thus, atrial arrhythmias can cause thromboembolic events resulting in cerebrovascular accidents, whilst ventricular arrhythmias predispose to sudden cardiac death. By contrast, arrhythmias in the GI tract are usually not life-threatening and perhaps this is the reason that they are much less well characterized. However, recent studies have implicated GI arrhythmogenesis with a number of motility disorders, which are associated with significant morbidity. The aim of this article is to compare and contrast the electrophysiological mechanisms of arrhythmogenesis in both systems, drawing analogies to shed light on the GI aspects. This is followed by a discussion on the clinical relevance as exemplified by GI motility disorders and molecular targets for future therapy.

## Ionic contributions to electrical activity

Smooth muscle cells of the GI tract generate slow waves, whereas cardiomyocytes in the heart produce action potentials (APs); both types of electrical activity are dependent upon ionic conductances across the cell membranes. The morphology of these waveforms dependent on the cell type and location in the respective specialized conduction systems. Thus, slow waves by gastric cells are triangular with rapid depolarization and repolarization phases. Slow waves of the small and large intestinal smooth muscle cells have an initial depolarizing phase generated by the pacemaker cells, interstitial cells of Cajal of the myenteric plexus (ICC-MY) (Dickens et al., 1999), and a second phase mediated by ICC within the smooth muscle (ICC-IM) (Bauer et al., 1985; Dickens et al., 2001). Superimposed upon these slow waves are regenerative Ca^2+^ spikes, which only develops when the membrane potential is above a threshold; these spikes are intrinsic to the smooth muscle cells (Lee et al., 1999; Suzuki and Hirst, 1999; Lammers and Slack, 2001). Cardiac APs have a rapid upstroke, rapid repolarization and a plateau phase. The reader is directed to these articles here for a review of the ionic currents mediating GI slow waves and cardiac APs (Lammers et al., 2009; Tse et al., 2016c). Both systems show features of restitution, where the duration of electrical activity shortens in response to higher pacing rates. Thus, slow waves in the gastric antrum normally discharges at a frequency of 1–2 cycles per minute (cpm) (Bauer et al., 1985; Publicover and Sanders, 1986). Upon a higher rate of extrinsic stimulation, it can exhibit waves at 7 cpm (Sarna and Daniel, 1973). This can be explained by restitution mechanisms that result from shortening or abolishing the plateau phase (Publicover and Sanders, 1986). Similarly, cardiac restitution is responsible for normal shortening of APD observed in response to faster heart rates, and is thought to be an adaptive mechanism for preserving diastole at these rates.

## Arrhythmogenic mechanisms

Both cardiac and gastrointestinal arrhythmias can be classified into non-reentrant and reentrant mechanisms (Table 1).

**Table 1:** **Arrhythmogenic mechanisms in the GI and cardiovascular systems can be divided into non-reentrant and reentrant activity**.

**Classification**	**Mechanism**	**Sub-types**	**Clinical relevance**	**References**
Non-reentrant	Enhanced pacemaker activity	–	GI: Gastroparesis, intestinal infection, inflammation and mitochondrial dysfunction	Der et al., 2000; O'Grady et al., 2011, 2012; Scheffer and Smout, 2011; Wu et al., 2013
		–	Cardiac: increased sympathetic tone, hypovolaemia, ischaemia, electrolyte disturbances	Jalife et al., 2009; Tse, 2015
	Triggered activity	Second potentials (GI)	Tachygastria	Daniel and Chapman, 1963; Suzuki and Hirst, 1999; Lammers and Slack, 2001; Qian et al., 2003; Lammers et al., 2008
		Early afterdepolarizations (cardiac)	Long QT syndromes, heart failure	Weiss et al., 2010; Maruyama et al., 2011
		Delayed afterdepolarizations (cardiac)	Ca^2+^ overload Catecholaminergic polymorphic ventricular tachycardia (CPVT), heart failure	Priori et al., 2001; Nam et al., 2005
Reentrant	Obstacle	Anatomical (GI and cardiac)	GI: circumferential reentry	Sinha et al., 2002; Angeli et al., 2013
			Cardiac: AV nodal reentrant tachycardia, AV reentrant tachycardia and pre-excitation syndromes, post-myocardial infarction, fibrosis in cardiomyopathies, myocarditis, cardio-metabolic disorders	Wong et al., 2013; Vassiliou et al., 2014; Baksi et al., 2015; Tse et al., 2015a,b; Tse et al., 2016a
		Functional (GI and cardiac)	GI: double-loop	Gullikson et al., 1980; Stoddard et al., 1981; Kim et al., 1987; Lammers et al., 2012; Angeli et al., 2013
			Cardiac: spiral and scroll wave, figure-of-eight, torsade de pointes	Allessie et al., 1973, 1975, 1976, 1977, 1989; Smeets et al., 1986; Rensma et al., 1988
	No obstacle	Reflection (cardiac)	Ischaemia	Antzelevitch et al., 1980; Antzelevitch and Moe, 1981; Rozanski et al., 1984; Lukas and Antzelevitch, 1989; Auerbach et al., 2011; Tung, 2011
		Phase 2 (cardiac)	Ischaemia, Ca^2+^ overload, Brugada syndrome	Kuo et al., 1983; Di Diego and Antzelevitch, 1993; Lukas and Antzelevitch, 1996; Shimizu et al., 2005

### Non-reentrant activity

**Figure 1:**
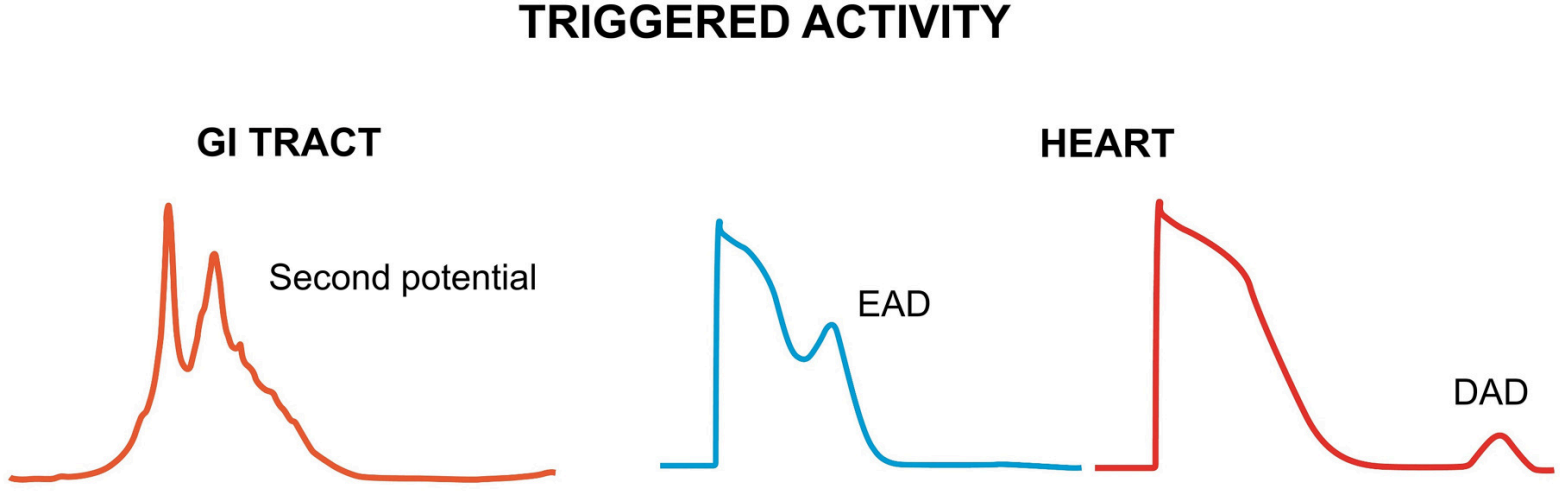
**Triggered activity can result from second potentials in the GI tract (left) or afterdepolarizations in the heart (right)**. Second potentials may be due to Ca^2+^ entry from the extracellular space or Ca^2+^ release from the endoplasmic reticulum. Early afterdepolarizations (EADs) are due to reactivation of L-type Ca^2+^ channels or Na^+^-Ca^2+^ exchanger (NCX). Delayed afterdepolarizations (DADs) develop during Ca^2+^ overload, which activates Ca^2+^-sensitive channels: non-selective cationic channel, NCX and calcium-activated chloride channel.

Non-reentrant activity refers to aberrant initiation due to either enhanced automaticity or triggered activity. Enhanced pacemaker activity in the heart can arise from a depolarizing shift of the maximum diastolic potential, a hyperpolarizing shift of the threshold potential or a faster rate of rise of the spontaneous depolarization (Jalife et al., 2009). In the GI tract, it has been observed in the human stomach (O'Grady et al., 2011, 2012), and the small intestine during inflammation, infection and mitochondrial disease (Der et al., 2000; Scheffer and Smout, 2011; Wu et al., 2013). By contrast, triggered activity refers to activity initiated by the preceding electrical activity (Figure 1). In the heart, it is due to early or delayed afterdepolarization phenomena (EADs and DADs, respectively), which are secondary depolarization events occurring before the subsequent AP (Cranefield, 1977; January et al., 1991), which can initiate arrhythmias (Tse, 2015). EADs are typically generated when the repolarization phase of the cardiac AP is prolonged, leading to reactivation of the L-type Ca^2+^ channels (*I*_Ca_) (January and Riddle, 1989) or activation of the Na^+^-Ca^2+^ exchanger (*I*_NCX_) secondary to spontaneous Ca^2+^ release from the sarcoplasmic reticulum (Szabo et al., 1994). DADs are associated with Ca^2+^ overload, which activates the following Ca^2+^-sensitive currents: the non-selective cationic current, *I*_NS_, the sodium-calcium exchange current, *I*_NCX_, and the calcium-activated chloride current, *I*_Cl, Ca_, which together constitute the transient inward current (*I*_TI_) (Guinamard et al., 2004). These afterdepolarizations are analogous to “second potentials” that could generate the ectopic beats observed in the GI tract (Qian et al., 2003). However, the mechanism underlying their generation is different. Increased automaticity here is related to increased stretch, enhanced by acetylcholine and inhibited by adrenaline (Daniel and Chapman, 1963). Their ionic contributions are yet to be determined, but could potentially involve Ca^2+^ entry from the extracellular space or Ca^2+^ release from the endoplasmic reticulum (Suzuki and Hirst, 1999; Lammers and Slack, 2001). Premature slow waves, which presumably arise from such secondary potentials, precede and may be a prerequisite for the initiation of tachygastria (Lammers et al., 2008).

### Reentry

**Figure 2:**
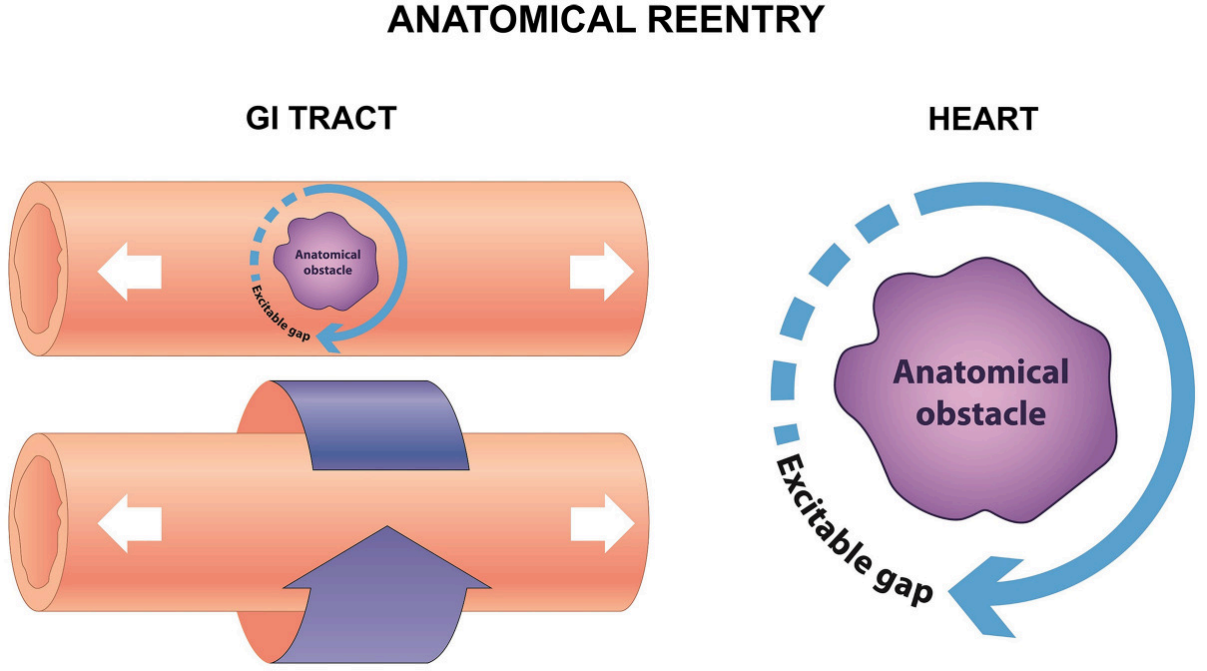
**Anatomical reentry in the GI tract can take place in the serosal surface, or around the circumference (left)**. In the heart, reentry can similarly take place around an anatomical obstacle, which may be a fibrotic scar, or areas of fibrosis **(right)**.

**Figure 3:**
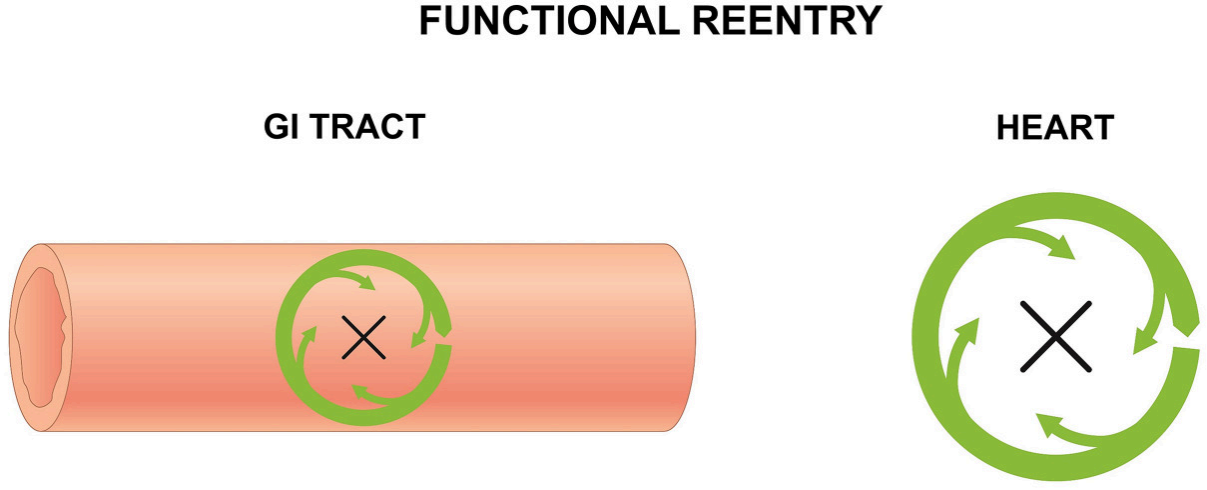
**Functional reentry in the GI tract (left) and the heart (right) involves circular activity around a central refractory obstacle**. This may arise from centripetal electrotonic forces that continuously provide subthreshold depolarization to the core, rendering it inexcitable, or from premature activation of the tissue concerned leading to absolute or relative refractoriness.

Reentry is a frequently encountered mechanism and occurs when an impulse fails to extinguish itself and re-excites a region that has recovered from refractoriness. In the heart, it can take place in the presence of an obstacle (circus-type), or in the absence of an obstacle (reflection or phase 2 reentry). Three requirements for circus-type reentry are reduced conduction velocity (CV), unidirectional conduction block and an obstacle around which the AP can circulate. This obstacle can be a permanent anatomical abnormality (anatomical reentry) (Figure 2), but can also involve a functional core of refractory tissue that arises dynamically (functional reentry) (Figure 3; Garrey, 1914).

There are a number of similarities between reentry occurring in the GI tract and the heart: initiation of a premature beat precede reentry, reentry can be non-sustained or sustained. Additionally, anisotropic conduction is important in both systems in reentry (Angeli et al., 2013). However, several differences are observed (Lammers et al., 2008). Firstly, gastric tachyarrhythmia occurs at a much lower frequency of 10–15 cycles per minute, whereas ventricular tachyarrhythmia typically occurs at rates between 100 and 250 beats per minute (bpm). Secondly, unidirectional conduction block is a prerequisite of circus-type reentry (Allessie et al., 1976, 1977; Lammers et al., 1990), but this is not the case in tachygastria as shown by electrograms recorded from the canine stomach using a multi-electrode array (Lammers et al., 2008).

#### Anatomical reentry

Circus-type reentry involving an anatomical obstacle was first demonstrated by the ring model using disks made from sub-umbrella tissue of a jellyfish (Mayer, 1906). Mayer made the following observations. The disks were paralyzed when they were separated from their sense organs. They do not pulsate in seawater, but did so when ring-like cuts were made from the tissue. Upon mechanical stimulation, the disks then showed “rhythmical pulsations so regular and sustained as to recall the movement of clockwork.” Later, Mines used a ring-like preparation of the tortoise heart, demonstrating that it was possible to initiate circus-type re-entry by electrical stimulation (Mines, 1913). He noted that when an excitation wave has a high CV and a long duration, the whole circuit would be excited at the same time, causing the excitation to die out. By contrast, when the wave has slow CV and a short ERP, the tissue ahead of the excitation wave would recover from refractoriness and can therefore be re-excited, resulting in circus-type re-entry. Mines predicted “a circulating excitation of this type may be responsible for some cases of paroxysmal tachycardia as observed clinically.” He was the first to formulate the three criteria for circus-type reentry mentioned above. It was later recognized that conduction of the excitation must be sufficiently slow to allow the tissue ahead in the circuit to recover from refractoriness so that it can be re-excited. It is useful to describe this excitation as a propagating wave (Weiss et al., 2005), with a wavefront that represents action potential depolarization, and a tail that represents repolarization (Weiss et al., 2000) with the assumption that APD is equal to the effective refractory period (ERP) (Tse et al., 2016i). The length of this excitation wave (λ) is given by CV × ERP (Wiener and Rosenblueth, 1946), and must be smaller than the length of the circuit in order for re-entry to be successful. Thus, reduced and increased λ is associated with greater and lesser likelihood of circus-type reentry, respectively (Smeets et al., 1986; Vaidya et al., 1999; Osadchii and Olesen, 2009; Osadchii et al., 2009, 2010; Osadchii, 2010, 2012a,b, 2014a,b, 2016; Tse et al., 2012, 2016b,c,d,e,f,g,h,j; Tse and Yan, 2016; Tse, 2016a,b,c).

Anatomical reentry is relevant in different types of tachyarrhythmias, such as AV nodal reentrant tachycardia, AV reentrant tachycardia and pre-excitation syndromes including Wolff-Parkinson-White Syndrome. It can be also the mechanism underlying atrial and ventricular tachycardia, where the AP wave circulates around a fixed fibrotic scar, such as post-myocardial infarction (Sinha et al., 2002). Moreover, micro-reentry around areas of fibrosis, which is observed in conditions such as cardiomyopathies, myocarditis and cardio-metabolic disorders of hypertension and diabetes mellitus (Wong et al., 2013; Vassiliou et al., 2014; Baksi et al., 2015; Tse et al., 2015a,b, 2016a). Anatomical reentry involving a fixed pathway has also been observed in the small intestine: re-entrant activity propagating around the circumference has been termed circumferential reentry (Angeli et al., 2013), which is analogous to the anatomical reentry (Allessie et al., 1977). In both cases, this is a fixed circuit whose length is determined by the perimeter of the anatomical obstacle, with an excitable gap between the depolarization wavefront and the repolarization tail. The revolution time is inversely proportional to the CV.

#### Functional reentry

For functional reentry without an anatomical obstacle, seminal experiments in rabbit atrial preparations provided its direct evidence in support of Garrey's prediction. Allessie applied electrical stimulation at the center of the atrial preparation and found that electrical activation elicited by regular stimuli spread normally throughout the atrial tissue (Allessie et al., 1973). Contrastingly, premature stimuli elicited electrical activity that only propagated in the direction of shortened ERPs and at a reduced CV. Spatial dispersion in the refractory periods (Allessie et al., 1976) was responsible for unidirectional block of the premature AP (Allessie et al., 1975). To explain the lack of activity in this core, it was proposed that center of the circle was held above threshold by the electrotonic influences of the depolarization wavefront propagating centripetally, which rendered it inexcitable. The AP would continue to revolve around this functional core of refractory tissue. Subsequent experiments utilizing transmembrane potential recordings led to the development of the leading circle model (Allessie et al., 1977). The circuit is defined entirely by the electrophysiological properties of the tissue. The smallest circuit permitting successful re-entry, called the leading circle, is one in which the circulating wavefront can just re-excite the tissue ahead that is still in its relative refractory period. A variation of functional reentry termed spiral wave reentry was described later (Krinsky, 1966). A spiral wave is a two-dimensional wave of excitation emitted by a self-organizing source of functional reentrant activity, termed a rotor. The three-dimensional equivalent of a spiral wave is a scroll wave.

Spiral waves were described earlier in the Belousov–Zhabotinsky chemical reaction, in which cerium catalyzes the malonic acid oxidation by bromate (Belousov, 1958; Zaikin and Zhabotinsky, 1970). The ratio of cerium (IV) to cerium (III) undergoes repeated temporal oscillations, producing spiral waves with alternating colors (Müller et al., 1985; Epstein, 2006). Later, spiral waves were reproduced in theoretical models of cardiac tissue (Moe et al., 1964; Courtemanche and Winfree, 1991; Leon et al., 1994) and demonstrated in thin slices of epicardial muscle using a potentiometric dye, whose spectral properties are altered by voltage (Salzberg et al., 1973). Previous experiments have demonstrated an excitable phase singularity, although it remains non-excited and can act as a functional obstacle around which the spiral wave can travel (Ikeda et al., 1996). Spiral waves are not fixed in space but can drift (Pertsov et al., 1993). This is accompanied by a Doppler effect, in which the frequency of excitation at a given measurement site depends on its location relative to the drifting spiral wave (Davidenko et al., 1992). Therefore, the sites anterior to the wave are excited faster than those posterior to the wave. Such a mechanism may underlie torsade de pointes (Dessertenne, 1966), whereby two widely separated foci discharging at different frequencies were suggested to underlie periodic torsion of the QRS axis.

Functional reentry in the GI tract can have analogous mechanisms (Gullikson et al., 1980; Stoddard et al., 1981; Kim et al., 1987; Lammers et al., 2012; Angeli et al., 2013). In the stomach, functional reentry can take a circular route (O'Grady et al., 2011) or have a double loop morphology, consisting of two wavefronts traveling in opposite directions (Lammers et al., 2008). The latter is similar to the cardiac figure-of-eight reentry generated by two counter-rotating spiral waves separated by a small distance (El-Sherif et al., 1981). Functional reentry has also been observed in the small intestine (Lammers et al., 2012; Angeli et al., 2013), which is analogous to Allessie's leading circle model of reentrant tachycardia in the atria with the following similarities (Allessie et al., 1977). Firstly, the length of the circuit is determined by electrophysiological rather than anatomical characteristics. Secondly, the dimensions of the circuits are variable rather than fixed. Thirdly, the depolarization front and the repolarization tail are in close proximity to each other, and so there is only a partially excitable gap between the two. Fourthly, the center of the circuit contains excitable rather than inexcitable tissue, which would permit termination of the re-entrant tachycardia if an impulse shorts the circuit by crossing the circle. Finally, the time for one rotation is inversely proportional to the RP of the tissue rather than to the CV of the wave. As pointed out, these circuits can meander along the tissue and are more unstable than anatomical reentrant circuits (Lammers, 2013). This supports previous modeling studies suggesting that self-sustaining spiral waves can be generated in anisotropic smooth muscle syncytium in the intestines (Miftahof, 2005).

## Autonomic modulation

Coumel originally proposed a triads of conditions necessary for arrhythmogenesis, which are trigger, substrate and modulating factors (Coumel et al., 1967, 1978; Coumel, 1993). In both systems, arrhythmias are susceptible to autonomic modulation (Smeets et al., 1986; El-Sherif et al., 1987; Ouyang et al., 2015). In the heart, parasympathomimetic agents such as acetylcholine reduces CV, APD and ERP, thereby decreasing the excitation wavelength to promote reentry (Smeets et al., 1986; Oliveira et al., 2011). In the presence of sympathomimetic agents such as noradrenaline, the Ca^2+^ transient increased (Bers, 2002a,b), and forward activation of NCX, with consequent EADs and triggered activity (Patterson et al., 2006). Sympathetic activation in long QT syndromes and catecholaminergic polymorphic ventricular tachycardia can exacerbate ventricular arrhythmias (Shen and Zipes, 2014). This may be due to increased heterogeneities in repolarization and refractoriness, thereby producing a favorable substrate for reentry. Autonomic dysfunction, particularly affecting vagal nerves, is known to result in GI motility disorders in diabetes mellitus (Feldman and Schiller, 1983). Slow wave arrhythmias in the small intestine mediated by hyperglycaemia is likely the result of higher sympathetic compared to parasympathetic activity (Ouyang et al., 2015). Interestingly, diabetic rats have a higher likelihood of developing functional reentry in the small intestine compared to the control rats (Lammers et al., 2012). The underlying cause is unclear, abnormalities in the enteric nervous system or the smooth muscle itself may be affected but autonomic dysfunction can well play an important role.

## Clinical relevance

The question remains, even if arrhythmias occur in the GI tract, are they clinically significant? To answer this, the following evidence should be considered. Gastric tachy- and brady-arrhythmias have been associated with gastroparesis (Bortolotti et al., 1990), in which reduced CV of slow waves has been observed (O'Grady et al., 2012). They also appear to be predictive of dyspeptic symptoms in systemic sclerosis (McNearney et al., 2009). Gastric tachyarrhythmias can be observed following administration of opiate drugs, after anesthesia or post-operatively (Stoddard et al., 1981). Anesthetic agents can act on Ca^2+^ channels directly (Ahn and Karaki, 1988), thereby leading to abnormal slow wave propagation and reentrant arrhythmias. Unexplained nausea and vomiting involves recurrent arrhythmias with abnormal wave propagation and higher frequency in the distal stomach, as demonstrated by gastric serosal electrophysiological study (Abell et al., 2009). Intestinal arrhythmias occur in diabetes mellitus (Lammers et al., 2012; Ouyang et al., 2015) and mesenteric ischemia (Seidel et al., 1999; Irimia and Wikswo, 2008) and may play a role in post-operative ileus, as suggested previously (Angeli et al., 2013).

However, only limited evidence exists on the mechanisms of arrhythmias occurring in these situations, but theoretical considerations suggest reentry playing a key role. Thus, spiral waves are inducible in the myocardium or intestinal smooth muscle because of intrinsic electrical heterogeneities and anisotropic properties (Miftahof, 2005) and their formation would be made more favorable in the above pathological conditions, which increase tissue heterogeneity and anisotropy (Gizzi et al., 2010). Alternatively, inflammation could result in loss of ICC-MY activity, suggesting that pacemaker activity is impaired (Yanagida et al., 2007; Gizzi et al., 2010). A better understanding of electrophysiology is key to developing effective treatment for these motility disorders. For example, almost all anti-arrhythmic agents in the heart are modulators of ion channels, which can also be targeted in the GI tract.

Bradyarrhythmias, although not discussed in this review, are also observed in many GI pathologies. Thus, the use of opiate drugs can abolish slow waves or lead to irregular patterns of slow waves, termed amyogenesia and dysmyogenesia, respectively (Sarna and Otterson, 1990). Other causes, where loss of GI pacemaker cells is observed, include achalasia (Chen et al., 2013), gastroparesis (O'Grady et al., 2012), functional dyspepsia (Jung et al., 2012), Hirschsprung disease (Yamataka et al., 1995), and slow transit constipation (Lyford et al., 2002).

Irritable bowel syndrome, a triad of altered bowel habits, bloating and abdominal pain without an organic cause (Sinagra et al., 2016) with either a diarrhea- or constipation- predominant phenotype, is a chronic debilitating relapsing and remitting condition. Loss of ICC-MY (Eshraghian and Eshraghian, 2011), Na^+^ channel mutations (Saito et al., 2009) and altered microbiota profile (Tana et al., 2010; Ng et al., 2013) increasing the intestinal Cl^−^ channel activity have been demonstrated (Chang and Talley, 2010). These changes could lead to impaired initiation of slow wave activity in the intestines. Furthermore, these abnormalities are accompanied by alterations in ICC-MY network and electrophysiological remodeling (Akbarali et al., 2010) caused by chronic inflammation (Der et al., 2000), and could conceivably serve as favorable substrates for reentrant arrhythmogenesis. Interestingly, clinical evidence does not support the notion that autonomic dysfunction plays a role in the symptoms associated with gastrointestinal motility disorders such as chronic dyspepsia or constipation (Vazeou et al., 2004).

Recently, a new syndrome characterized by Chronic Atrial and Intestinal Dysrhythmia, termed CAID syndrome, has been discovered, in which features of both sick sinus syndrome (SSS) (of alternating bradycardia-tachycardia, Chen et al., 2016b) and chronic intestinal pseudo-obstruction (CIPO) are observed (Chetaille et al., 2014). In CAID, mutation in SGOL1, a component of the cohesin complex, was the underlying cause. Both SSS and CIPO are caused by pacemaker dysfunction: SSS can be caused by loss-of-function mutations in the SCN5A gene encoding for the sodium channel, whereas CIPO is caused by loss of the ICC-MY (Feldstein et al., 2003; Struijs et al., 2008). Moreover, atrial fibrillation gut syndrome (AFGS) was used to describe reduced gastrointestinal motility, e.g., gastroparesis, following radiofrequency catheter ablation for atrial fibrillation (Lee and Lee, 2014). This may arise from vagus nerve injury from electrical injury used for ablation.

## Future treatment options and concluding remarks

Improved understanding of the abnormal electrophysiology underlying GI motility disorders can lead to the development of more effective treatment options. In terms of pharmacotherapy, ion channels represent attractive targets. For example, functional constipation or constipation-predominant IBS can be managed by the chloride channel protein 2 agonist, lubiprostone (Camilleri et al., 2006; Andresen et al., 2007), whereas functional diarrhea or diarrhea -predominant IBS can be managed by its inhibitor crofelemer (Manabe et al., 2010; Yeo et al., 2013). For intervention, analogous to cardiac pacing for heart blocks, gastric electrical stimulation can be used in severe cases of gastroparesis (Abrahamsson, 2007). Similarly, colonic electrical stimulation can potentially be used for chronic functional constipation or constipation-predominant IBS (Chen et al., 2016a). Ablation has been used extensively for the management of atrial fibrillation, but it role in gastrointestinal arrhythmogenesis is unclear.

Despite the importance of GI electrophysiology, it is considerably underdeveloped compared to the cardiac electrophysiology, which is a sub-specialty of cardiology (O'Grady et al., 2014). A deeper understanding of the molecular basis and physiological mechanisms underlying GI motility disorders will enable the development of better diagnostic and therapeutic tools and the advancement of this field.

## Author contributions

GT: Design of manuscript; drafted and critically revised the manuscript for important intellectual content; preparation of figures. EL: Acquired and interpreted primary research papers; critically revised the manuscript for important intellectual content; preparation of figures. AL: Analyzed and interpreted primary research papers; critically revised the manuscript for important intellectual content. BY: Analyzed and interpreted primary research papers; critically revised the manuscript for important intellectual content. SW: Analyzed and interpreted primary research papers; critically revised the manuscript for important intellectual content.

### Conflict of interest statement

The authors declare that the research was conducted in the absence of any commercial or financial relationships that could be construed as a potential conflict of interest.

## References

[B1] Abell T. L. Familoni B. Voeller G. Werkman R. Dean P. Waters B.. (2009). Electrophysiologic, morphologic, and serologic features of chronic unexplained nausea and vomiting: lessons learned from 121 consecutive patients. Surgery 145, 476–485. 10.1016/j.surg.2008.12.006 19375605

[B2] Abrahamsson H. (2007). Treatment options for patients with severe gastroparesis. Gut 56, 877–883. 10.1136/gut.2005.078121 17519490 PMC1954884

[B3] Ahn H. Y. Karaki H. (1988). Inhibitory effects of procaine on contraction and calcium movement in vascular and intestinal smooth muscles. Br. J. Pharmacol. 94, 789–796. 10.1111/j.1476-5381.1988.tb11590.x 3179612 PMC1854047

[B4] Akbarali H. I. Hawkins E. G. Ross G. R. Kang M. (2010). Ion channel remodeling in gastrointestinal inflammation. Neurogastroenterol. Motil. 22, 1045–1055. 10.1111/j.1365-2982.2010.01560.x 20618833 PMC2939949

[B5] Allessie M. A. Bonke F. I. Schopman F. J. (1973). Circus movement in rabbit atrial muscle as a mechanism of tachycardia. Circ. Res. 33, 54–62. 10.1161/01.RES.33.1.54 4765700

[B6] Allessie M. A. Bonke F. I. Schopman F. J. (1975). The mechanism of supraventricular tachycardia induced by a single premature beat in the isolated left atrium of the rabbit. I. Circus movement as a consequence of unidirectional block of the premature impulse. Recent Adv. Stud. Cardiac Struct. Metab. 5, 303–308. 1188162

[B7] Allessie M. A. Bonke F. I. Schopman F. J. (1976). Circus movement in rabbit atrial muscle as a mechanism of tachycardia. II. The role of nonuniform recovery of excitability in the occurrence of unidirectional block, as studied with multiple microelectrodes. Circ. Res. 39, 168–177. 10.1161/01.RES.39.2.168 939001

[B8] Allessie M. A. Bonke F. I. Schopman F. J. (1977). Circus movement in rabbit atrial muscle as a mechanism of tachycardia. III. The “leading circle” concept: a new model of circus movement in cardiac tissue without the involvement of an anatomical obstacle. Circ. Res. 41, 9–18. 10.1161/01.RES.41.1.9 862147

[B9] Allessie M. A. Schalij M. J. Kirchhof C. J. Boersma L. Huybers M. Hollen J. (1989). Experimental electrophysiology and arrhythmogenicity. Anisotropy and ventricular tachycardia. Eur. Heart J. 10, 2–8. 10.1093/eurheartj/10.suppl_E.2 2680500

[B10] Andresen V. Camilleri M. Busciglio I. A. Grudell A. Burton D. McKinzie S.. (2007). Effect of 5 days linaclotide on transit and bowel function in females with constipation-predominant irritable bowel syndrome. Gastroenterology 133, 761–768. 10.1053/j.gastro.2007.06.067 17854590

[B11] Angeli T. R. O'Grady G. Du P. Paskaranandavadivel N. Pullan A. J. Bissett I. P.. (2013). Circumferential and functional re-entry of *in vivo* slow-wave activity in the porcine small intestine. Neurogastroenterol. Motil. 25, e304–e314. 10.1111/nmo.12085 23489929 PMC3781238

[B12] Antzelevitch C. Jalife J. Moe G. K. (1980). Characteristics of reflection as a mechanism of reentrant arrhythmias and its relationship to parasystole. Circulation 61, 182–191. 10.1161/01.CIR.61.1.182 7349933

[B13] Antzelevitch C. Moe G. K. (1981). Electrotonically mediated delayed conduction and reentry in relation to “slow responses” in mammalian ventricular conducting tissue. Circ. Res. 49, 1129–1139. 10.1161/01.RES.49.5.1129 7296780

[B14] Auerbach D. S. Grzda K. R. Furspan P. B. Sato P. Y. Mironov S. Jalife J. (2011). Structural heterogeneity promotes triggered activity, reflection and arrhythmogenesis in cardiomyocyte monolayers. J. Physiol. 589, 2363–2381. 10.1113/jphysiol.2010.200576 21486795 PMC3098708

[B15] Baksi A. J. Kanaganayagam G. S. Prasad S. K. (2015). Arrhythmias in viral myocarditis and pericarditis. Card. Electrophysiol. Clin. 7, 269–281. 10.1016/j.ccep.2015.03.009 26002391

[B16] Bauer A. J. Reed J. B. Sanders K. M. (1985). Slow wave heterogeneity within the circular muscle of the canine gastric antrum. J. Physiol. 366, 221–232. 10.1113/jphysiol.1985.sp015793 4057090 PMC1193028

[B17] Belousov B. P. (1958). A periodically occuring reaction and its mechanism (Russian title). Sborn. Referat. Radiats. Med. 145–147.

[B18] Bers D. M. (2002a). Calcium and cardiac rhythms: physiological and pathophysiological. Circ. Res. 90, 14–17. 11786512

[B19] Bers D. M. (2002b). Cardiac excitation-contraction coupling. Nature 415, 198–205. 10.1038/415198a 11805843

[B20] Bortolotti M. Sarti P. Barbara L. Brunelli F. (1990). Gastric myoelectric activity in patients with chronic idiopathic gastroparesis. Neurogastroenterol. Motil. 2, 104–108. 10.1111/j.1365-2982.1990.tb00015.x

[B21] Camilleri M. Bharucha A. E. Ueno R. Burton D. Thomforde G. M. Baxter K.. (2006). Effect of a selective chloride channel activator, lubiprostone, on gastrointestinal transit, gastric sensory, and motor functions in healthy volunteers. Am. J. Physiol. Gastrointest. Liver Physiol. 290, G942–G947. 10.1152/ajpgi.00264.2005 16603730

[B22] Chang J. Y. Talley N. J. (2010). Current and emerging therapies in irritable bowel syndrome: from pathophysiology to treatment. Trends Pharmacol. Sci. 31, 326–334. 10.1016/j.tips.2010.04.008 20554042

[B23] Chen J. H. Wang X. Y. Liu L. W. Yu W. Yu Y. Zhao L.. (2013). On the origin of rhythmic contractile activity of the esophagus in early achalasia, a clinical case study. Front. Neurosci. 7:77. 10.3389/fnins.2013.00077 23734090 PMC3659367

[B24] Chen S. Liu L. Guo X. Yao S. Li Y. Chen S.. (2016a). Effects of colonic electrical stimulation using different individual parameter patterns and stimulation sites on gastrointestinal transit time, defecation, and food intake. Int. J. Colorectal Dis. 31, 429–437. 10.1007/s00384-015-2457-6 26607906

[B25] Chen Z. Sun B. Tse G. Jiang J. Xu W. (2016b). Reversibility of both sinus node dysfunction and reduced HCN4 mRNA expression level in an atrial tachycardia pacing model of tachycardia-bradycardia syndrome in rabbit hearts. Int. J. Clin. Exp. Pathol.

[B26] Chetaille P. Preuss C. Burkhard S. Côté J.-M. Houde C. Castilloux J.. (2014). Mutations in SGOL1 cause a novel cohesinopathy affecting heart and gut rhythm. Nat. Genet. 46, 1245–1249. 10.1038/ng.3113 25282101

[B27] Coumel P. (1993). Cardiac arrhythmias and the autonomic nervous system. J. Cardiovasc. Electrophysiol. 4, 338–355. 10.1111/j.1540-8167.1993.tb01235.x 8269304

[B28] Coumel P. Cabrol C. Fabiato A. Gourgon R. Slama R. (1967). Tachycardiamente par rythme reciproque. Arch. Mal. Coeur Vaiss. 60, 1830–1864.

[B29] Coumel P. Fidelle J. Lucet V. Attuel P. Bouvrain Y. (1978). Catecholamine-induced severe ventricular arrhythmias with Adams-Stokes syndrome in children: report of four cases. Br. Heart J. 40, 28–37.

[B30] Courtemanche M. Winfree A. T. (1991). Re-entrant rotating waves in a Beeler-Reuter based model of two-dimensional cardiac electrical activity. Int. J. Bifurcation Chaos 1, 431–444. 10.1142/S0218127491000336

[B31] Cranefield P. F. (1977). Action potentials, afterpotentials, and arrhythmias. Circ. Res. 41, 415–423. 10.1161/01.RES.41.4.415 409566

[B32] Daniel E. E. Chapman K. M. (1963). Electrical activity of the gastrointestinal tract as an indication of mechanical activity. Am. J. Dig. Dis. 8, 54–102. 10.1007/BF02233560 14024909

[B33] Davidenko J. M. Pertsov A. V. Salomonsz R. Baxter W. Jalife J. (1992). Stationary and drifting spiral waves of excitation in isolated cardiac muscle. Nature 255, 349–351. 10.1038/355349a0 1731248

[B34] Der T. Bercik P. Donnelly G. Jackson T. Berezin I. Collins S. M.. (2000). Interstitial cells of cajal and inflammation-induced motor dysfunction in the mouse small intestine. Gastroenterology 119, 1590–1599. 10.1053/gast.2000.20221 11113080

[B35] Dessertenne F. (1966). La tachycardie ventriculaire a deux foyers opposes variable. Arch. Mal. Coeur 56, 263–272. 4956181

[B36] Dickens E. J. Edwards F. R. Hirst G. D. (2001). Selective knockout of intramuscular interstitial cells reveals their role in the generation of slow waves in mouse stomach. J. Physiol. 531, 827–833. 10.1111/j.1469-7793.2001.0827h.x 11251061 PMC2278487

[B37] Dickens E. J. Hirst G. D. Tomita T. (1999). Identification of rhythmically active cells in guinea-pig stomach. J. Physiol. 514 (Pt 2), 515–531. 10.1111/j.1469-7793.1999.515ae.x 9852332 PMC2269070

[B38] Di Diego J. M. Antzelevitch C. (1993). Pinacidil-induced electrical heterogeneity and extrasystolic activity in canine ventricular tissues. Does activation of ATP-regulated potassium current promote phase 2 reentry? Circulation 88, 1177–1189. 10.1161/01.CIR.88.3.1177 7689041

[B39] El-Sherif N. Gough W. B. Restivo M. (1987). Reentrant ventricular arrhythmias in the late myocardial infarction period: 14. Mechanisms of resetting, entrainment, acceleration, or termination of reentrant tachycardia by programmed electrical stimulation. Pacing Clin. Electrophysiol. 10, 341–371. 10.1111/j.1540-8159.1987.tb05974.x 2437540

[B40] El-Sherif N. Smith R. A. Evans K. (1981). Canine ventricular arrhythmias in the late myocardial infarction period. 8. Epicardial mapping of reentrant circuits. Circ. Res. 49, 255–265. 10.1161/01.RES.49.1.255 7237696

[B41] Epstein I. R. (2006). Predicting complex biology with simple chemistry. Proc. Natl. Acad. Sci. U.S.A. 103, 15727–15728. 10.1073/pnas.0608026103 17043211 PMC1635071

[B42] Eshraghian A. Eshraghian H. (2011). Interstitial cells of Cajal: a novel hypothesis for the pathophysiology of irritable bowel syndrome. Can. J. Gastroenterol. 25, 277–279. 10.1155/2011/478370 21647464 PMC3115010

[B43] Feldman M. Schiller L. R. (1983). Disorders of gastrointestinal motility associated with diabetes mellitus. Ann. Intern. Med. 98, 378–384. 10.7326/0003-4819-98-3-378 6402969

[B44] Feldstein A. E. Miller S. M. El-Youssef M. Rodeberg D. Lindor N. M. Burgart L. J.. (2003). Chronic intestinal pseudoobstruction associated with altered interstitial cells of cajal networks. J. Pediatr. Gastroenterol. Nutr. 36, 492–497. 10.1097/00005176-200304000-00016 12658043

[B45] Garrey W. E. (1914). The nature of fibrillary contraction of the heart: its relation to tissue mass and form. Am. J. Physiol. 33, 397–414.

[B46] Gizzi A. Cherubini C. Migliori S. Alloni R. Portuesi R. Filippi S. (2010). On the electrical intestine turbulence induced by temperature changes. Phys. Biol. 7, 16011. 10.1088/1478-3975/7/1/016011 20147777

[B47] Guinamard R. Chatelier A. Demion M. Potreau D. Patri S. Rahmati M.. (2004). Functional characterization of a Ca^(2+)^-activated non-selective cation channel in human atrial cardiomyocytes. J. Physiol. 558, 75–83. 10.1113/jphysiol.2004.063974 15121803 PMC1664929

[B48] Gullikson G. W. Okuda H. Shimizu M. Bass P. (1980). Electrical arrhythmias in gastric antrum of the dog. Am. J. Physiol. 239, G59–G68. 7396005 10.1152/ajpgi.1980.239.1.G59

[B49] Ikeda T. Uchida T. Hough D. Lee J. J. Fishbein M. C. Mandel W. J.. (1996). Mechanism of spontaneous termination of functional reentry in isolated canine right atrium. Evidence for the presence of an excitable but nonexcited core. Circulation 94, 1962–1973. 10.1161/01.CIR.94.8.1962 8873675

[B50] Irimia A. Wikswo J. P. Jr. (2008). Gastrointestinal arrhythmias are associated with statistically significant fluctuations in systemic information dimension. Physiol. Meas. 29, N33–N40. 10.1088/0967-3334/29/5/N01 18427160 PMC7722964

[B51] Jalife J. Delmar M. Anumonwo J. Berenfeld O. Kalifa J. (eds.). (2009). Basic mechanisms of cardiac arrhythmias, in Basic Cardiac Electrophysiology for the Clinician, 2nd Edn (Wiley-Blackwell), 92–121.

[B52] January C. T. Chau V. Makielski J. C. (1991). Triggered activity in the heart: cellular mechanisms of early after-depolarizations. Eur. Heart J. 12, 4–9. 10.1093/eurheartj/12.suppl_F.4 1725155

[B53] January C. T. Riddle J. M. (1989). Early afterdepolarizations: mechanism of induction and block. A role for L-type Ca^2+^ current. Circ. Res. 64, 977–990. 10.1161/01.RES.64.5.977 2468430

[B54] Jung K. T. Park H. Kim J. H. Shin D. J. Joung B. Y. Lee M. H.. (2012). The relationship between gastric myoelectric activity and SCN5A mutation suggesting sodium channelopathy in patients with brugada syndrome and functional dyspepsia - a pilot study. J. Neurogastroenterol. Motil. 18, 58–63. 10.5056/jnm.2012.18.1.58 22323988 PMC3271254

[B55] Kim C. H. Zinsmeister A. R. Malagelada J. R. (1987). Mechanisms of canine gastric dysrhythmia. Gastroenterology 92, 993–999. 3557006 10.1016/0016-5085(87)90975-9

[B56] Krinsky V. I. (1966). Spread of excitation in an homogeneous medium. Biophys. J. 11, 776–784.

[B57] Kuo C. S. Munakata K. Reddy C. P. Surawicz B. (1983). Characteristics and possible mechanism of ventricular arrhythmia dependent on the dispersion of action potential durations. Circulation 67, 1356–1367. 10.1161/01.CIR.67.6.1356 6851031

[B58] Lammers W. J. (2013). Arrhythmias in the gut. Neurogastroenterol. Motil. 25, 353–357. 10.1111/nmo.12116 23490042

[B59] Lammers W. J. E. P. Ver Donck L. Stephen B. Smets D. Schuurkes J. A. J. (2008). Focal activities and re-entrant propagations as mechanisms of gastric tachyarrhythmias. Gastroenterology 135, 1601–1611. 10.1053/j.gastro.2008.07.020 18713627

[B60] Lammers W. J. Schalij M. J. Kirchhof C. J. Allessie M. A. (1990). Quantification of spatial inhomogeneity in conduction and initiation of reentrant atrial arrhythmias. Am. J. Physiol. 259, H1254–H1263. 1699438 10.1152/ajpheart.1990.259.4.H1254

[B61] Lammers W. J. Slack J. R. (2001). Of slow waves and spike patches. News Physiol. Sci. 16, 138–144. 11443235 10.1152/physiologyonline.2001.16.3.138

[B62] Lammers W. J. Stephen B. Karam S. M. (2012). Functional reentry and circus movement arrhythmias in the small intestine of normal and diabetic rats. Am. J. Physiol. Gastrointest. Liver Physiol. 302, G684–G689. 10.1152/ajpgi.00332.2011 22207580

[B63] Lammers W. J. Ver Donck L. Stephen B. Smets D. Schuurkes J. A. (2009). Origin and propagation of the slow wave in the canine stomach: the outlines of a gastric conduction system. Am. J. Physiol. Gastrointest. Liver Physiol. 296, G1200–G1210. 10.1152/ajpgi.90581.2008 19359425

[B64] Lee D. S. Lee S. J. (2014). Severe gastroparesis following radiofrequency catheter ablation for atrial fibrillation: suggestion for diagnosis, treatment, and device for gastroparesis after RFCA. Case Rep. Gastrointest. Med. 2014, 6. 10.1155/2014/923637 25614842 PMC4295156

[B65] Lee J. C. Thuneberg L. Berezin I. Huizinga J. D. (1999). Generation of slow waves in membrane potential is an intrinsic property of interstitial cells of Cajal. Am. J. Physiol. 277, G409–G423. 10444456 10.1152/ajpgi.1999.277.2.G409

[B66] Leon L. J. Roberge F. A. Vinet A. (1994). Simulation of two-dimensional anisotropic cardiac reentry: effects of the wavelength on the reentry characteristics. Ann. Biomed. Eng. 22, 592–609. 10.1007/BF02368286 7872570

[B67] Lukas A. Antzelevitch C. (1989). Reflected reentry, delayed conduction, and electrotonic inhibition in segmentally depressed atrial tissues. Can. J. Physiol. Pharmacol. 67, 757–764. 10.1139/y89-121 2766107

[B68] Lukas A. Antzelevitch C. (1996). Phase 2 reentry as a mechanism of initiation of circus movement reentry in canine epicardium exposed to simulated ischemia. Cardiovasc. Res. 32, 593–603. 10.1016/0008-6363(96)00115-0 8881520

[B69] Lyford G. L. He C. L. Soffer E. Hull T. L. Strong S. A. Senagore A. J.. (2002). Pan-colonic decrease in interstitial cells of Cajal in patients with slow transit constipation. Gut 51, 496–501. 10.1136/gut.51.4.496 12235070 PMC1773407

[B70] Manabe N. Rao A. S. Wong B. S. Camilleri M. (2010). Emerging pharmacologic therapies for irritable bowel syndrome. Curr. Gastroenterol. Rep. 12, 408–416. 10.1007/s11894-010-0124-1 20694841

[B71] Maruyama M. Lin S. F. Xie Y. Chua S. K. Joung B. Han S.. (2011). Genesis of phase 3 early afterdepolarizations and triggered activity in acquired long-QT syndrome. Circ. Arrhythm. Electrophysiol. 4, 103–111. 10.1161/CIRCEP.110.959064 21078812 PMC3045276

[B72] Mayer A. G. (1906). Rhythmical Pulsation in Scyphomedusae. Washington, DC: Carnegie Institute of Washington.

[B73] McNearney T. A. Sallam H. S. Hunnicutt S. E. Doshi D. Wollaston D. E. Mayes M. D.. (2009). Gastric slow waves, gastrointestinal symptoms and peptides in systemic sclerosis patients. Neurogastroenterol. Motil. 21, e1269–e1120. 10.1111/j.1365-2982.2009.01350.x 19566588 PMC3176740

[B74] Miftahof R. (2005). A novel intrinsic wave phenomenon in low excitable biological media, in Mechanisms, Symbols, and Models Underlying Cognition: First International Work-Conference on the Interplay between Natural and Artificial Computation, IWINAC 2005, Las Palmas, Canary Islands, Spain, June 15-18, 2005, Proceedings, Part I, eds Mira J. Álvarez J. R. (Berlin; Heidelberg: Springer Berlin Heidelberg), 38–47.

[B75] Mines G. R. (1913). On dynamic equilibrium in the heart. J. Physiol. 46, 349–383. 10.1113/jphysiol.1913.sp001596 16993210 PMC1420430

[B76] Moe G. K. Rheinboldt W. C. Abildskov J. A. (1964). A computer model of atrial fibrillation. Am. Heart J. 67, 200–220. 10.1016/0002-8703(64)90371-0 14118488

[B77] Müller S. C. Plesser T. Hess B. (1985). The structure of the core of the spiral wave in the belousov-zhabotinskii reaction. Science 230, 661–663. 10.1126/science.230.4726.661 17797290

[B78] Nam G. B. Burashnikov A. Antzelevitch C. (2005). Cellular mechanisms underlying the development of catecholaminergic ventricular tachycardia. Circulation 111, 2727–2733. 10.1161/CIRCULATIONAHA.104.479295 15911700 PMC1474839

[B79] Ng S. C. Lam E. F. Lam T. T. Chan Y. Law W. Tse P. C.. (2013). Effect of probiotic bacteria on the intestinal microbiota in irritable bowel syndrome. J. Gastroenterol. Hepatol. 28, 1624–1631. 10.1111/jgh.12306 23800182

[B80] O'Grady G. Angeli T. R. Du P. Lahr C. Lammers W. J. Windsor J. A.. (2012). Abnormal initiation and conduction of slow-wave activity in gastroparesis, defined by high-resolution electrical mapping. Gastroenterology 143, 589-598.e1–3. 10.1053/j.gastro.2012.05.036 22643349 PMC3429650

[B81] O'Grady G. Egbuji J. U. Du P. Lammers W. J. Cheng L. K. Windsor J. A.. (2011). High-resolution spatial analysis of slow wave initiation and conduction in porcine gastric dysrhythmia. Neurogastroenterol. Motil. 23, e345–e355. 10.1111/j.1365-2982.2011.01739.x 21714831 PMC3156377

[B82] O'Grady G. Wang T. H. Du P. Angeli T. Lammers W. J. Cheng L. K. (2014). Recent progress in gastric arrhythmia: pathophysiology, clinical significance and future horizons. Clin. Exp. Pharmacol. Physiol. 41, 854–862. 10.1111/1440-1681.12288 25115692 PMC4359928

[B83] Oliveira M. da Silva N. Cunha P. Ramos R. Marques F. Santos S.. (2011). Effects of acute autonomic modulation on atrial conduction delay and local electrograms duration in paroxysmal atrial fibrillation. Int. J. Cardiol. 149, 290–295. 10.1016/j.ijcard.2010.02.006 20299115

[B84] Osadchii O. E. (2010). Mechanisms of hypokalemia-induced ventricular arrhythmogenicity. Fundam. Clin. Pharmacol. 24, 547–559. 10.1111/j.1472-8206.2010.00835.x 20584206

[B85] Osadchii O. E. (2012a). Effects of ventricular pacing protocol on electrical restitution assessments in guinea-pig heart. Exp. Physiol. 97, 807–821. 10.1113/expphysiol.2012.065219 22447974

[B86] Osadchii O. E. (2012b). Flecainide-induced proarrhythmia is attributed to abnormal changes in repolarization and refractoriness in perfused guinea-pig heart. J. Cardiovasc. Pharmacol. 60, 456–466. 10.1097/FJC.0b013e31826b86cf 22932706

[B87] Osadchii O. E. (2014a). Impact of hypokalemia on electromechanical window, excitation wavelength and repolarization gradients in guinea-pig and rabbit hearts. PLoS ONE 9:e105599. 10.1371/journal.pone.0105599 25141124 PMC4139393

[B88] Osadchii O. E. (2014b). Impaired epicardial activation-repolarization coupling contributes to the proarrhythmic effects of hypokalaemia and dofetilide in guinea pig ventricles. Acta Physiol. 211, 48–60. 10.1111/apha.12259 24533513

[B89] Osadchii O. E. (2016). Flecainide attenuates rate adaptation of ventricular repolarization in guinea-pig heart. Scand. Cardiovasc. J. 50, 28–35. 10.3109/14017431.2015.1099721 26402340

[B90] Osadchii O. E. Bentzen B. H. Olesen S. P. (2009). Chamber-specific effects of hypokalaemia on ventricular arrhythmogenicity in isolated, perfused guinea-pig heart. Exp. Physiol. 94, 434–446. 10.1113/expphysiol.2008.045567 19151074

[B91] Osadchii O. E. Larsen A. P. Olesen S. P. (2010). Predictive value of electrical restitution in hypokalemia-induced ventricular arrhythmogenicity. Am. J. Physiol. Heart Circ. Physiol. 298, H210–H220. 10.1152/ajpheart.00695.2009 19897712

[B92] Osadchii O. E. Olesen S. P. (2009). Electrophysiological determinants of hypokalaemia-induced arrhythmogenicity in the guinea-pig heart. Acta Physiol. 197, 273–287. 10.1111/j.1748-1716.2009.02028.x 19656123

[B93] Ouyang X. Li S. Foreman R. Farber J. Lin L. Yin J.. (2015). Hyperglycemia-induced small intestinal dysrhythmias attributed to sympathovagal imbalance in normal and diabetic rats. Neurogastroenterol. Motil. 27, 406–415. 10.1111/nmo.12506 25630445

[B94] Patterson E. Lazzara R. Szabo B. Liu H. Tang D. Li Y. H.. (2006). Sodium-calcium exchange initiated by the Ca^2+^ transient: an arrhythmia trigger within pulmonary veins. J. Am. Coll. Cardiol. 47, 1196–1206. 10.1016/j.jacc.2005.12.023 16545652

[B95] Pertsov A. M. Davidenko J. M. Salomonsz R. Baxter W. T. Jalife J. (1993). Spiral waves of excitation underlie reentrant activity in isolated cardiac muscle. Circ. Res. 72, 631–650. 10.1161/01.RES.72.3.631 8431989

[B96] Priori S. G. Napolitano C. Tiso N. Memmi M. Vignati G. Bloise R.. (2001). Mutations in the cardiac ryanodine receptor gene (hRyR2) underlie catecholaminergic polymorphic ventricular tachycardia. Circulation 103, 196–200. 10.1161/01.CIR.103.2.196 11208676

[B97] Publicover N. G. Sanders K. M. (1986). Effects of frequency on the wave form of propagated slow waves in canine gastric antral muscle. J. Physiol. 371, 179–189. 10.1113/jphysiol.1986.sp015967 3701649 PMC1192716

[B98] Qian L. W. Pasricha P. J. Chen J. D. (2003). Origins and patterns of spontaneous and drug-induced canine gastric myoelectrical dysrhythmia. Dig. Dis. Sci. 48, 508–515. 10.1023/A:1022532515172 12757162

[B99] Rensma P. L. Allessie M. A. Lammers W. J. Bonke F. I. Schalij M. J. (1988). Length of excitation wave and susceptibility to reentrant atrial arrhythmias in normal conscious dogs. Circ. Res. 62, 395–410. 10.1161/01.RES.62.2.395 3338122

[B100] Rozanski G. J. Jalifé J. Moe G. K. (1984). Reflected reentry in nonhomogeneous ventricular muscle as a mechanism of cardiac arrhythmias. Circulation 69, 163–173. 10.1161/01.CIR.69.1.163 6689641

[B101] Saito Y. A. Strege P. R. Tester D. J. Locke G. R. III Talley N. J. Bernard C. E.. (2009). Sodium channel mutation in irritable bowel syndrome: evidence for an ion channelopathy. Am. J. Physiol. Gastrointest. Liver Physiol. 296, G211–G218. 10.1152/ajpgi.90571.2008 19056759 PMC2643921

[B102] Salzberg B. M. Davila H. V. Cohen L. B. (1973). Optical recording of impulses in individual neurones of an invertebrate central nervous system. Nature 246, 508–509. 10.1038/246508a0 4357630

[B103] Sarna S. K. Daniel E. E. (1973). Electrical stimulation of gastric electrical control activity. Am. J. Physiol. 225, 125–131. 4714390 10.1152/ajplegacy.1973.225.1.125

[B104] Sarna S. K. Otterson M. F. (1990). Small intestinal amyogenesia and dysmyogenesia induced by morphine and loperamide. Am. J. Physiol. 258, G282–G289. 1968317 10.1152/ajpgi.1990.258.2.G282

[B105] Scheffer R. C. Smout A. J. (2011). Tachyduodenia in mitochondrial neurogastrointestinal encephalomyopathy. Neurogastroenterol. Motil. 23, 408–410. 10.1111/j.1365-2982.2011.01698.x 21481099

[B106] Seidel S. A. Hegde S. S. Bradshaw L. A. Ladipo J. K. Richards W. O. (1999). Intestinal tachyarrhythmias during small bowel ischemia. Am. J. Physiol. 277, G993–G999. 10564105 10.1152/ajpgi.1999.277.5.G993

[B107] Shen M. J. Zipes D. P. (2014). Role of the autonomic nervous system in modulating cardiac arrhythmias. Circ. Res. 114, 1004–1021. 10.1161/CIRCRESAHA.113.302549 24625726

[B108] Shimizu W. Aiba T. Kamakura S. (2005). Mechanisms of disease: current understanding and future challenges in Brugada syndrome. Nat. Clin. Pract. Cardiovasc. Med. 2, 408–414. 10.1038/ncpcardio0268 16119703

[B109] Sinagra E. Pompei G. Tomasello G. Cappello F. Morreale G. C. Amvrosiadis G.. (2016). Inflammation in irritable bowel syndrome: myth or new treatment target? World J. Gastroenterol. 22, 2242–2255. 10.3748/wjg.v22.i7.2242 26900287 PMC4734999

[B110] Sinha S. Stein K. M. Christini D. J. (2002). Critical role of inhomogeneities in pacing termination of cardiac reentry. Chaos 12, 893–902. 10.1063/1.1501176 12779614

[B111] Smeets J. L. Allessie M. A. Lammers W. J. Bonke F. I. Hollen J. (1986). The wavelength of the cardiac impulse and reentrant arrhythmias in isolated rabbit atrium. The role of heart rate, autonomic transmitters, temperature, and potassium. Circ. Res. 58, 96–108. 10.1161/01.RES.58.1.96 3943157

[B112] Stoddard C. J. Smallwood R. H. Duthie H. L. (1981). Electrical arrhythmias in the human stomach. Gut 22, 705–712. 10.1136/gut.22.9.705 7028578 PMC1419876

[B113] Struijs M.-C. Diamond I. R. Pencharz P. B. Chang K. T. E. Viero S. Langer J. C.. (2008). Absence of the interstitial cells of Cajal in a child with chronic pseudoobstruction. J. Pediatr. Surg. 43, e25–e29. 10.1016/j.jpedsurg.2008.09.017 19040916

[B114] Suzuki H. Hirst G. D. S. (1999). Regenerative potentials evoked in circular smooth muscle of the antral region of guinea-pig stomach. J. Physiol. 517, 563–573. 10.1111/j.1469-7793.1999.0563t.x 10332102 PMC2269361

[B115] Szabo B. Sweidan R. Rajagopalan C. V. Lazzara R. (1994). Role of Na^+^:Ca^2+^ exchange current in Cs^(+)^-induced early afterdepolarizations in Purkinje fibers. J. Cardiovasc. Electrophysiol. 5, 933–944. 10.1111/j.1540-8167.1994.tb01133.x 7889233

[B116] Tana C. Umesaki Y. Imaoka A. Handa T. Kanazawa M. Fukudo S. (2010). Altered profiles of intestinal microbiota and organic acids may be the origin of symptoms in irritable bowel syndrome. Neurogastroenterol. Motil. 22, e512–e115. 10.1111/j.1365-2982.2009.01427.x 19903265

[B117] Tse G. (2015). Mechanisms of cardiac arrhythmias. J. Arrhythm. 32, 75–81. 10.1016/j.joa.2015.11.003 27092186 PMC4823581

[B118] Tse G. (2016a). Both transmural dispersion of repolarization and transmural dispersion of refractoriness are poor predictors of arrhythmogenicity: a role for the index of Cardiac Electrophysiological Balance (QT/QRS)? J. Geriatr. Cardiol. 10.11909/j.issn.1671-5411.2016.09.007 PMC5122509 27899948

[B119] Tse G. (2016b). Novel conduction-repolarization indices for the stratification of arrhythmic risk. J. Geriatr. Cardiol. 10.11909/j.issn.1671-5411.2016.09.008 PMC5122508 27899947

[B120] Tse G. (2016c). (Tpeak-Tend)/QRS and (Tpeak-Tend)/(QT x QRS): novel markers for predicting arrhythmic risk in Brugada syndrome. Europace. 10.1093/europace/euw194 28431069

[B121] Tse G. Ali A. Alpendurada F. Prasad S. Raphael C. E. Vassiliou V. (2015a). Tuberculous constrictive pericarditis. Res Cardiovasc. Med. 4:e29614. 10.5812/cardiovascmed.29614 26793674 PMC4707979

[B122] Tse G. Ali A. Prasad S. K. Vassiliou V. Raphael C. E. (2015b). Atypical case of post-partum cardiomyopathy: an overlap syndrome with arrhythmogenic right ventricular cardiomyopathy? BJR Case Rep. 1:20150182 10.1259/bjrcr.20150182 PMC6159128 30363137

[B123] Tse G. Hothi S. S. Grace A. A. Huang C. L. (2012). Ventricular arrhythmogenesis following slowed conduction in heptanol-treated, Langendorff-perfused mouse hearts. J. Physiol. Sci. 62, 79–92. 10.1007/s12576-011-0187-2 22219003 PMC10717265

[B124] Tse G. Lai E. T. Tse V. Yeo J. M. (2016a). Molecular and electrophysiological mechanisms underlying cardiac arrhythmogenesis in diabetes mellitus. J. Diabetes Res. 10.1155/2016/2848759 PMC5011530 27642609

[B125] Tse G. Lai E. T. Yeo J. M. Yan B. P. (2016b). Electrophysiological mechanisms of Bayés syndrome: insights from clinical and mouse studies. Front. Physiol. 7:188 10.3389/fphys.2016.00188 PMC4886053 27303306

[B126] Tse G. Lai T. H. Yeo J. M. Tse V. Wong S. H. (2016c). Mechanisms of electrical activation and conduction in the gastrointestinal system: lessons from cardiac electrophysiology. Front. Physiol. 7:182 10.3389/fphys.2016.00182 PMC4885840 27303305

[B127] Tse G. Sun B. Wong S. T. Tse V. Yeo J. M. (2016d). Ventricular anti-arrhythmic effects of hypercalcaemia treatment in hyperkalaemic, Langendorff-perfused mouse hearts. Biomed. Rep. 10.3892/br.2016.577. [Epub ahead of print]. PMC4998139 27588173

[B128] Tse G. Tse V. Yeo J. M. (2016e). Ventricular anti-arrhythmic effects of heptanol in hypokalaemic, Langendorff-perfused mouse hearts. Biomed Rep. 4, 313–324. 10.3892/br.2016.577 26998268 PMC4774402

[B129] Tse G. Tse V. Yeo J. M. Sun B. (2016f). Atrial anti-arrhythmic effects of heptanol in Langendorff-perfused mouse hearts. PLoS ONE 11:e0148858. 10.1371/journal.pone.0148858 26872148 PMC4752503

[B130] Tse G. Wong S. T. Tse V. Yeo J. M. (2016g). Depolarization vs. repolarization: what is the mechanism of ventricular arrhythmogenesis underlying sodium channel haploinsufficiency in mouse hearts? Acta Physiol. 10.1111/apha.12694. [Epub ahead of print]. 27272698

[B131] Tse G. Wong S. T. Tse V. Yeo J. M. (2016h). Determination of action potential wavelength restitution in Scn5a+/− mouse hearts modelling human Brugada syndrome. J. Physiol. 10.11909/j.issn.1671-5411.2017.09.011 PMC5641650 29056961

[B132] Tse G. Wong S. T. Tse V. Yeo J. M. (2016i). Monophasic action potential recordings: which is the recording electrode? J. Basic Clin. Physiol. Pharmacol. 10.1515/jbcpp-2016-0007. [Epub ahead of print]. 27135622

[B133] Tse G. Wong S. T. Tse V. Yeo J. M. (2016j). Restitution analysis of alternans using dynamic pacing and its comparison with S1S2 restitution in heptanol-treated, hypokalaemic Langendorff-perfused mouse hearts. Biomed Rep. 4, 673–680. 10.3892/br.2016.659 27284405 PMC4887808

[B134] Tse G. Wong S. T. Tse V. Lee Y. T. Lin H. Y. Yeo J. M. (2016k). Cardiac dynamics: alternans and arrhythmogenesis. J. Arrhythm. 10.1016/j.joa.2016.02.009. [Epub ahead of print]. 27761166 PMC5063258

[B135] Tse G. Yan B. P. (2016). Novel arrhythmic risk markers incorporating QRS dispersion: QRSd x (Tpeak-Tend)/QRS and QRSd x (Tpeak-Tend)/(QT x QRS). Ann. Noninvasive. Electrocardiol. 10.1111/anec.12397 PMC6931740 27535213

[B136] Tse G. Yeo J. M. (2015). Conduction abnormalities and ventricular arrhythmogenesis: the roles of sodium channels and gap junctions. Int. J. Cardiol. Heart Vasc. 9, 75–82. 10.1016/j.ijcha.2015.10.003 26839915 PMC4695916

[B137] Tung L. (2011). Expanding on forty years of reflection. J. Physiol. 589, 2107–2108. 10.1113/jphysiol.2011.209239 21532028 PMC3098685

[B138] Vaidya D. Morley G. E. Samie F. H. Jalife J. (1999). Reentry and fibrillation in the mouse heart. A challenge to the critical mass hypothesis. Circ. Res. 85, 174–181. 10.1161/01.RES.85.2.174 10417399

[B139] Vassiliou V. Chin C. Perperoglou A. Tse G. Ali A. Raphael C. (2014). 93 Ejection fraction by cardiovascular magnetic resonance predicts adverse outcomes post aortic valve replacement. Heart 100, A53–A54. 10.1136/heartjnl-2014-306118.93

[B140] Vazeou A. Papadopoulou A. Papadimitriou A. Kitsou E. Stathatos M. Bartsocas C. S. (2004). Autonomic neuropathy and gastrointestinal motility disorders in children and adolescents with type 1 diabetes mellitus. J. Pediatr. Gastroenterol. Nutr. 38, 61–65. 10.1097/00005176-200401000-00014 14676596

[B141] Weiss J. N. Chen P. S. Qu Z. Karagueuzian H. S. Garfinkel A. (2000). Ventricular fibrillation: how do we stop the waves from breaking? Circ. Res. 87, 1103–1107. 10.1161/01.RES.87.12.1103 11110766

[B142] Weiss J. N. Garfinkel A. Karagueuzian H. S. Chen P. S. Qu Z. (2010). Early afterdepolarizations and cardiac arrhythmias. Heart Rhythm 7, 1891–1899. 10.1016/j.hrthm.2010.09.017 20868774 PMC3005298

[B143] Weiss J. N. Qu Z. Chen P. S. Lin S. F. Karagueuzian H. S. Hayashi H.. (2005). The dynamics of cardiac fibrillation. Circulation 112, 1232–1240. 10.1161/CIRCULATIONAHA.104.529545 16116073

[B144] Wiener N. Rosenblueth A. (1946). The mathematical formulation of the problem of conduction of impulses in a network of connected excitable elements, specifically in cardiac muscle. Arch. Inst. Cardiol. Mex. 16, 205–265. 20245817

[B145] Wong W. T. Tian X. Y. Huang Y. (2013). Endothelial dysfunction in diabetes and hypertension: cross talk in RAS, BMP4, and ROS-dependent COX-2-derived prostanoids. J. Cardiovasc. Pharmacol. 61, 204–214. 10.1097/FJC.0b013e31827fe46e 23232839

[B146] Wu R. Y. Pasyk M. Wang B. Forsythe P. Bienenstock J. Mao Y. K.. (2013). Spatiotemporal maps reveal regional differences in the effects on gut motility for Lactobacillus reuteri and rhamnosus strains. Neurogastroenterol. Motil. 25, e205–e214. 10.1111/nmo.12072 23316914

[B147] Yamataka A. Kato Y. Tibboel D. Murata Y. Sueyoshi N. Fujimoto T.. (1995). A lack of intestinal pacemaker (c-kit) in aganglionic bowel of patients with Hirschsprung's disease. J. Pediatr. Surg. 30, 441–444. 10.1016/0022-3468(95)90051-9 7539078

[B148] Yanagida H. Sanders K. M. Ward S. M. (2007). Inactivation of inducible nitric oxide synthase protects intestinal pacemaker cells from postoperative damage. J. Physiol. 582, 755–765. 10.1113/jphysiol.2006.126482 17510193 PMC2075327

[B149] Yeo Q. M. Crutchley R. Cottreau J. Tucker A. Garey K. W. (2013). Crofelemer, a novel antisecretory agent approved for the treatment of HIV-associated diarrhea. Drugs Today 49, 239–252. 10.1358/dot.2013.49.4.1947253 23616951

[B150] Zaikin A. N. Zhabotinsky A. M. (1970). Concentration wave propagation in two-dimensional liquid-phase self-oscillating system. Nature 225, 535–537. 10.1038/225535b0 16056595

